# Oleuropein Regulates Bile Acid Metabolism via Modulating the Gut Microbiota, Thereby Alleviating DSS-Induced Ulcerative Colitis in Mice

**DOI:** 10.3390/foods14111863

**Published:** 2025-05-23

**Authors:** Rongxin Zang, Rui Zhou, Yaodong Li, Zhouliang Liu, Huihao Wu, Liping Lu, Hongwei Xu

**Affiliations:** 1 College of Life Science and Engineering, Northwest Minzu University, Lanzhou 730100, China; rxzang2000@163.com (R.Z.); zhour1222@163.com (R.Z.); lyaodong@163.com (Y.L.); a134063518367@163.com (Z.L.); 901030@126.com (L.L.); 2 Engineering Research Center of Key Technology and Industrialization of Cell-Based Vaccine, Ministry of Education, Lanzhou 730030, China; 3 Key Laboratory of Biotechnology and Bioengineering of State Ethnic Affairs Commission, Northwest Minzu University, Lanzhou 730030, China; wuhuihao99@163.com

**Keywords:** oleuropein, ulcerative colitis, nuclear factor kappa-B

## Abstract

The pathogenesis of ulcerative colitis (UC) involves genetic, immunological, and environmental factors as well as gut microbiota dysbiosis. As a natural antioxidant with various pharmacological activities widely present in Oleaceae plants, oleuropein (OLE) exhibits anti-inflammatory, anti-tumor, antiviral, hypoglycemic, and cardioprotective effects. It has been validated that OLE extracted from olive oil can ameliorate UC. However, it remains unclear if and how OLE modulates the gut microbiota in the alleviation of UC. Therefore, this study was conducted to explore the mechanisms for OLE to alleviate UC induced by dextran sulfate sodium (DSS), with the focus placed on its regulatory function in the gut microbiota. The results indicated that OLE mitigated DSS-induced UC by enhancing the intestinal barrier function, reshaping the gut microbiota, and modulating bile acid metabolism. The fecal microbiota transplantation (FMT) experiment results further confirmed that the protective effect of OLE against UC could be mediated by alterations in the gut microbiota and their metabolites induced by OLE. Additionally, OLE increased the abundance of *Lactobacillus* and certain bile acid metabolites in the colon, including hyodeoxycholic acid (HDCA). HDCA could upregulate the expression of ZO-1 and claudin-3, restoring intestinal barrier integrity. Simultaneously, HDCA could inhibit the activation of the nuclear factor kappa-B (NF-κB) signaling pathway in the colon and relieve colonic inflammation. Overall, it was corroborated that OLE alleviated DSS-induced UC by modulating the gut microbiota and altering bile acid metabolism.

## 1. Introduction

Ulcerative colitis (UC), a chronic inflammatory bowel disease (IBD), primarily affects the colon and rectum, distinguished by persistent mucosal inflammation and ulcer formation in the intestinal lining [1,2]. Characterized by chronic inflammation of the colonic mucosa and ulcer formation, UC can present some symptoms, such as diarrhea, abdominal pain, rectal bleeding, and weight loss [3]. In recent years, the incidence of UC has increased, thus imposing a significant public health burden worldwide [4]. Current therapeutic strategies for UC mainly rely on pharmacological interventions and surgical approaches to manage inflammation. Aminosalicylates and glucocorticoids serve as first-line treatments. However, aminosalicylates achieve symptom control in only 50% of patients with mild to moderate active disease, while glucocorticoids are associated with serious adverse effects such as hypertension, glaucoma, and osteoporosis when used long-term [5,6]. Immunosuppressants like thiopurines and calcineurin inhibitors can cause liver and kidney damage. Therefore, it is of great significance to identify alternative options for the treatment of UC.

In recent years, the impact of natural polyphenolic compounds on human health has received extensive attention. Studies have shown that antioxidants such as polyphenols and vitamins can protect intestinal cells from free radical damage and enhance immunity [7,8,9]. Meanwhile, polyphenolic substances in plants can also help patients to restore physiological functions and improve nutrient malabsorption caused by intestinal damage, playing a unique role in comprehensive treatment and enhancing therapeutic efficacy while improving patients’ quality of life [9]. Oleuropein (OLE), a natural polyphenolic compound derived from olive trees, has attracted significant attention in the food industry due to its diverse biological activities, including antioxidant, anti-inflammatory, antibacterial, and cardiovascular protective effects [7,10,11,12]. The natural product OLE has exhibited various pharmacological effects, including antioxidant properties in the treatment of atherosclerosis and diabetes and antimicrobial effects [7]. It has been revealed that OLE can inhibit the proliferation of tumor cells and promote tumor cell apoptosis through various mechanisms [8]. Additionally, it has been found that OLE can inhibit the metastasis of various malignant tumor cells, such as those found in breast cancer, lung cancer, and liver cancer [9,10,11]. Although several studies have demonstrated that oleuropein exerts a therapeutic effect in UC by alleviating oxidative stress and modulating inflammatory cytokines, the underlying molecular mechanisms through which it ameliorates UC remain unclear [5,6,7,8]. Therefore, further experimental investigations are warranted to elucidate the specific molecular pathways through which oleuropein exerts its therapeutic effects in UC treatment. This comprehensive study was designed to investigate the impacts of OLE on DSS-induced UC and clarify the underlying mechanistic pathways.

## 2. Materials and Methods

### 2.1. Animals and Experiments

**Figure 1:**
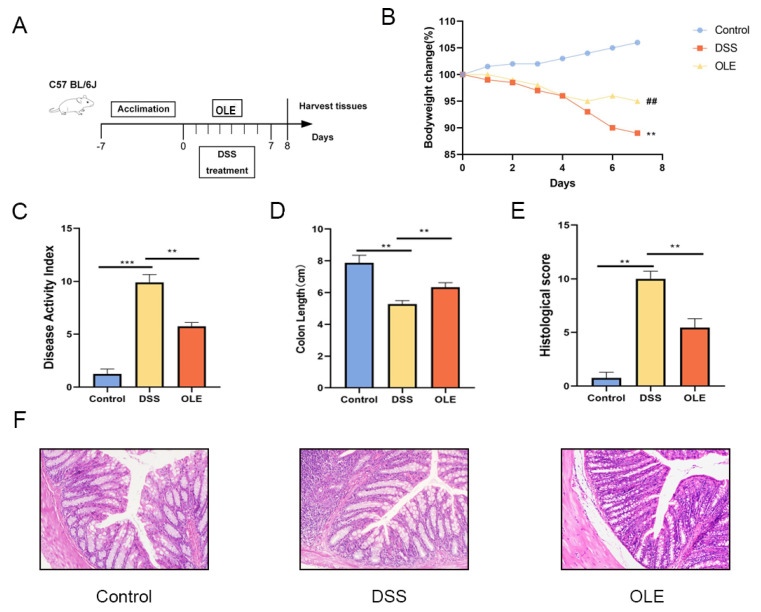
OLE treatment mitigated DSS-induced colitis in mice. (**A**) Schematic representation of the experimental protocol. (**B**) Percentage changes in body weight. (**C**) Disease activity index (DAI) scores. (**D**) Measurement of colon length. (**E**) Histopathological scoring results. (**F**) Colonic tissues from each group were prepared for histological analysis, with representative images showing structural alterations in mouse colons across different experimental groups. Data are presented as mean values ± standard deviation (*n* = 9). Statistical significance was denoted as ** *p* < 0.01, and *** *p* < 0.001 when compared with the DSS-treated group, ## *p* < 0.01 when OLE compared with the DSS-treated group.

In total, eighty-one 7-week-old C57BL/6 mice (18–22 g) were obtained from the Animal Experimental Center of Northwest Minzu University (Lanzhou, China). The mice were housed under specific pathogen-free (SPF) conditions with the following controlled environmental parameters: temperature of 20–24 °C, 50–60% relative humidity, a 12 h light/dark cycle, and air changes 10–15 times/h. OLE (Cat#HY-N0292) was purchased from MedChem Express (Shanghai, China). In the experiment involving the treatment of UC in mice with OLE, after the mice were acclimatized for 1 week, the male mice were randomly divided into three groups, namely, the control group, the DSS group, and the OLE group (*n* = 9). The preparation and administration of OLE were as follows: OLE was first dissolved in sterile dimethyl sulfoxide (DMSO), then diluted to the target concentration with phosphate-buffered saline (PBS; pH = 7.4) to ensure that the final DMSO volume fraction did not exceed 1%. The solution was sterilized by filtration through a 0.22 μm sterile membrane filter, freshly prepared, and stored in the dark. The control group and DSS-treated group were given the same volume of the PBS solution as a vehicle control. The mice in the DSS and OLE groups were provided with 3% (*w*/*v*) DSS in their drinking water for 7 days to induce colitis from Day 0. The mice in the control group were provided with sterile water throughout the study. The mice in the OLE group were fed with 20 mg/kg of OLE daily from the 1st day of DSS administration to the 7th day. The mice in the DSS group were provided with an equivalent volume of sterile PBS (Figure 1A).

In the FMT experiment, the male C57/BL mice were randomly divided into the following three groups. In the O-FMT and C-FMT groups, the control mice were fed with tap water supplemented with 3% (*w*/*v*) DSS for 7 days and treated with FMT for 1 week (*n* = 9). In the DSS group, the mice were fed with tap water supplemented with 3% DSS for 7 days (*n* = 9). In the O-FMT group, the OLE mice were used as donors, and they were fed with tap water supplemented with 3% (*w*/*v*) DSS for 7 days and treated with FMT for 1 week (*n* = 9). FMT was implemented as per the procedures in previous studies [4]. More specifically, fresh feces from each group were pooled, homogenized, and diluted with sterile saline to a final concentration of 50 mg/mL. Then, the mixed samples were centrifuged at 1000× *g* for 2 min and the supernatant was filtered through a 70 μm filter for FMT.

In the HDCA treatment experiment, the male C57/BL mice (7 weeks old) were randomly divided into the following three groups. In the control group (*n* = 9), the mice received 100 μL of PBS continuously for 1 week. In the DSS group (*n* = 9), the mice were provided with tap water supplemented with 3% (*w*/*v*) DSS for 7 days. In the HDCA group (*n* = 9), the mice were provided with tap water supplemented with 3% DSS for 7 days and then received 50 mg/kg HDCA.

### 2.2. Disease Activity Index (DAI) Analysis

Following the start of DSS administration, daily monitoring was conducted for the body weight and fecal status of all mice. DAI scores were documented according to the degree of weight loss using the following scoring criteria: 0 points for no weight loss; 1 point for 1–5% loss; 2 points for 5–10% loss; 3 points for 10–15% loss; and 4 points for loss exceeding 15%. Additionally, assessments were performed for fecal consistency (0 points for normal consistency, 2 points for loose stools, and 4 points for watery diarrhea) and rectal bleeding (0 points for no bleeding, 2 points for mild bleeding, and 4 points for obvious hemorrhage). The overall DAI score, ranging from 0 to 12, was calculated by summing the scores obtained from these three evaluation parameters [4].

### 2.3. Measurement of Pro-Inflammatory Cytokine Levels 

The colonic tissues of the mice were harvested and homogenized in PBS. Following homogenization, the tissue lysate was centrifuged at 12,000× *g* for 20 min at 4 °C, after which the supernatant was carefully collected. The levels of TNF-α, IL-1β, and IL-6 in the samples were quantified using ELISA kits (ELISA MAX Deluxe Sets; BioLegend, San Diego, CA, USA). Absorbance readings were obtained at 450 nm, with the background correction performed by subtracting the optical density measured at 570 nm.

### 2.4. Measurement of Oxidative Stress Levels 

Commercial kits (Nanjing Jiancheng Bioengineering Institute, Nanjing, China) were used to detect representative oxidoreductases, such as MPO, and MDA. The data could have been affected by the incubation time, incubation temperature, plate washing times, spectrophotometer wavelength, and nature of different antibodies.

### 2.5. Tissue Section Staining

The colonic distal tissues of the mice were fixed in a 4% formalin solution for 24 h, followed by embedding in paraffin. Then, the tissues were sectioned into slices of 5 µm in thickness and stained with hematoxylin and eosin (H&E) using a standard protocol. According to the established criteria, histological scoring was conducted based on the severity of damage and inflammatory infiltration in the mucosal layer, submucosal layer, muscularis mucosae, and serosal layer of colonic tissues. Finally, the total histopathological score for each mouse was calculated [5].

### 2.6. Western Blot Analysis

The protein was extracted from the colonic tissue of the mice using a protein extraction kit. The amount of protein extracted from each sample was determined using a BCA protein quantification assay. Specifically, the proteins were boiled in a 5× protein SDS-PAGE loading buffer and separated using 10% SDS-PAGE gel electrophoresis. Then, the protein in the SDS-PAGE was transferred onto a 0.2 µm PVDF membrane. After being blocked with bovine serum albumin overnight, the membrane was incubated with primary antibodies at 4 °C. The following day, the membrane was washed and then incubated with an HRP-conjugated secondary antibody at room temperature for 1 h. The signal was visualized using an enhanced chemiluminescence kit. The immunosignal intensity was quantified using ImageJ (version 1.8.0.112).

### 2.7. 16S rRNA Analysis of Mouse Fecal Microbiota

Genomic DNA extraction from fecal samples was performed using a QIAamp DNA Stool Mini Kit (Qiagen, Hilden, Germany), following the manufacturer’s standardized protocols. PCR product purification was subsequently carried out using an AxyPrep DNA Gel Extraction Kit (AxyPrep Biosciences, Union City, CA, USA). The library construction for sequencing utilized a NEXTFLEX^®^ Rapid DNA-Seq Kit, after which high-throughput sequencing was conducted using the Illumina MiSeq platform (Illumina, San Diego, CA, USA) according to the standard operating procedures provided by Majorbio Bio-pharma Technology Co., Ltd. (Shanghai, China). Raw 16SrRNA gene sequences underwent quality-control filtering using fastp (v0.20.0) and subsequent merging via FLASH (v1.2.7). Clustering into operational taxonomic units (OTUs) was achieved at a 97% similarity threshold using the UPARSE algorithm, with chimeric sequences identified and removed during this process. The taxonomic classification of OTUs was performed using the RDP Classifier (v2.2), assigning sequences to taxonomic ranks based on the 16SrRNA database with a confidence score ≥ 0.7. Alpha diversity metrics, including Chao1, Simpson, ACE, and Observed Species indices, were calculated to assess within-sample diversity. A beta diversity analysis, focusing on between-sample compositional differences, was performed via a principal coordinate analysis (PCoA) using Bray–Curtis dissimilarity matrices to visualize microbial community structures.

### 2.8. Gut Metabolite Assay

Following a previously reported method, fecal and colonic bile acids (BAs) were analyzed. Briefly, analyses were conducted using a Waters ACQUITY UPLC system coupled to a Waters XEVO TQ-S mass spectrometer equipped with an ESI source, operated via MassLynx 4.1 software (Waters, Milford, MA, USA). Chromatographic separation utilized a Waters ACQUITY BEH C18 column. Negative-mode raw data were processed using Waters TargetLynx Application Manager (v4.1) to generate calibration curves and quantify the BA concentrations in each sample.

### 2.9. Statistical Analysis

Statistical analyses were performed using SPSS 24.0 (IBM, Armonk, NY, USA). Intergroup comparisons across the three experimental groups were conducted via a one-way analysis of variance (ANOVA). Continuous data were presented as mean values ± standard deviation (SD). Statistical significance was defined as a *p*-value < 0.05.

## 3. Results

### 3.1. OLE Alleviated DSS-Induced Colitis in Mice

**Figure 2:**
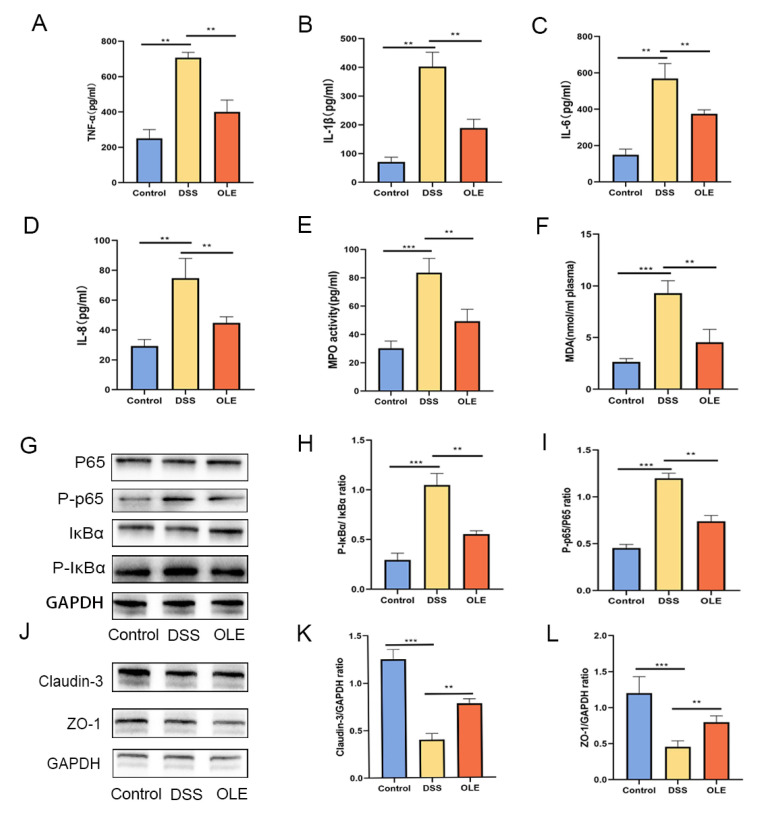
The effects of OLE on the expression levels of pro-inflammatory cytokines, oxidative stress markers, and tight-junction-associated proteins were examined. (**A**–**D**) Levels of TNF-α, IL-1β, IL-6, and IL-8 were measured, while (**E**,**F**) myeloperoxidase (MPO) and malondialdehyde (MDA) served as indicators of oxidative stress. OLE-mediated protection against colitis was associated with inhibition of the NF-κB signaling pathway, as shown in panels (**G**–**I**). Protein expression of the tight junction proteins claudin-3 and ZO-1 across the three experimental groups was assessed via Western blot analyses (**J**–**L**). All data represent the mean ± standard deviation of six independent samples (*n* = 6). Statistical significance was denoted as ** *p* < 0.01, and *** *p* < 0.001 when compared with the DSS-treated group.

To investigate the alleviating effect of OLE on DSS-induced colitis, the mice were provided with a 3% DSS solution for 7 consecutive days, followed by daily oral administration of 20 mg/kg body weight of OLE (Figure 1A). The body weight, DAI, colon length, and histological changes in the colon were evaluated in these mice (Figure 1B–F). Compared with the control group, the DAI significantly increased in the DSS group, while this trend was reversed in the OLE group (*p* < 0.05). The histological analysis results revealed that the DSS treatment induced obvious inflammatory cell infiltration and barrier damage in the colon compared with the control group, which was reduced by the addition of OLE (*p* < 0.05). Furthermore, mice in the OLE group exhibited lower levels of pro-inflammatory markers in the colon, including cytokines (Figure 2A–D) and oxidative stress markers (Figure 2E,F) (*p* < 0.05).

### 3.2. OLE Protected the Intestinal Mucosal Barrier in UC Mice and Inhibited the Expression of NF-κB Signaling

A further analysis revealed that OLE treatment significantly suppressed the expression of phosphorylated p65 and p-IκBα proteins in the NF-κB signaling pathway (*p* < 0.05) (Figure 2G–I). Relative to the control group, the DSS-treated group exhibited a reduced expression of tight junction (TJ) proteins ZO-1 and claudin-3. However, these alterations were reversed following OLE intervention, with statistically significant differences observed (*p* < 0.05) (Figure 2J–L).

### 3.3. The FMT of OLE Alleviated DSS-Induced Colitis in Mice

**Figure 3:**
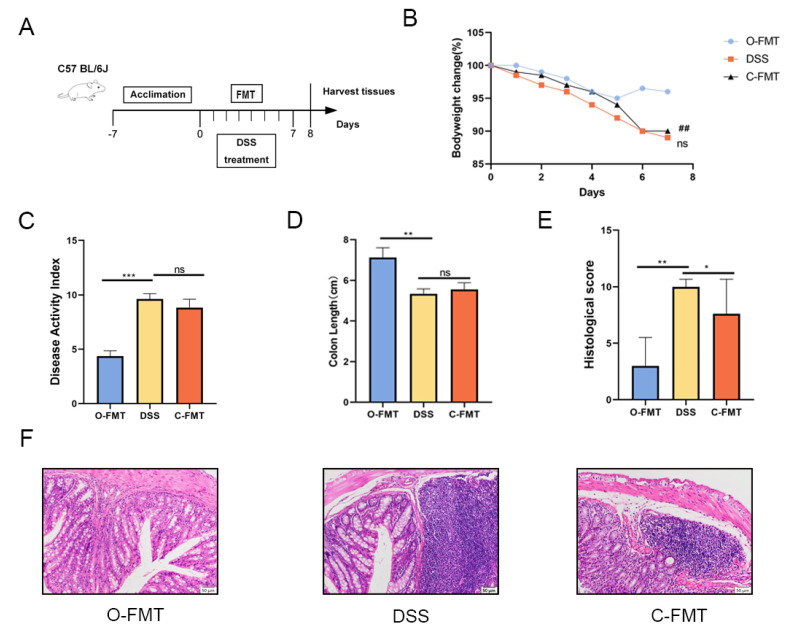
O-FMT treatment alleviated DSS-induced colitis in mice. (**A**) Schematic illustration of the experimental design. (**B**) Percentage variations in body weight over the observation period. (**C**) Evaluation of disease activity index (DAI) scores. (**D**) Measurement of colon length as a key morphological parameter. (**E**) Histopathological scoring outcomes reflecting colonic tissue damage. (**F**) Colonic samples from each group underwent histological processing, with representative images showing structural changes in mouse colons across different treatment groups. Data are presented as mean values ± standard deviation (*n* = 9). Statistical significance was denoted as * *p* < 0.05, ** *p* < 0.01, and *** *p* < 0.001 when compared with the DSS-treated group. ## *p* < 0.01 when OLE compared with the DSS-treated group.

**Figure 4:**
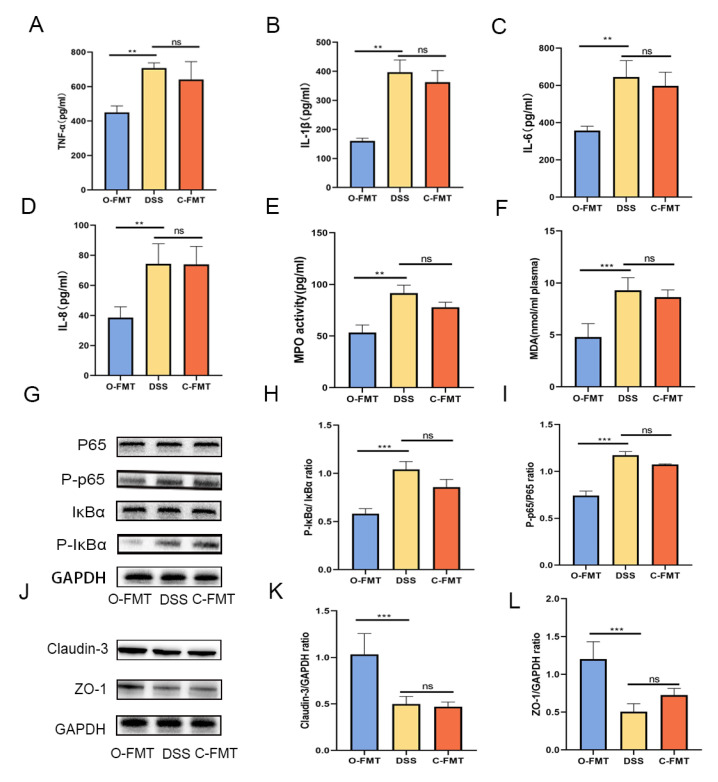
O-FMT modulated the expression of pro-inflammatory cytokines, oxidative stress markers, and tight junction proteins in DSS-induced colitis mice. (**A**–**D**) Quantification of TNF-α, IL-1β, IL-6, and IL-8 levels was performed, while (**E**,**F**) MPO and MDA were evaluated as indices of oxidative stress. Protection conferred by O-FMT against colitis was linked to suppression of the NF-κB signaling pathway, as depicted in panels (**G**–**I**). A Western blot analysis was employed to assess the protein expression of tight junction components claudin-3 and ZO-1 across the three experimental groups (**J**–**L**). All data are presented as mean values ± standard deviation based on six independent samples (*n* = 6). Statistical significance is indicated by ** *p* < 0.01, and *** *p* < 0.001 when compared with the DSS-treated group.

To verify the role of the gut microbiota in the protective effect of OLE against DSS-induced UC, FMT samples were collected from the control group and the OLE diet group (Figure 3A). Consistent with the above results, O-FMT intervention significantly alleviated DSS-induced colitis, which was proved by a significant decrease in DAI scores, weight loss, and colon shortening (Figure 3B–F). Additionally, O-FMT reduced pro-inflammatory markers in the colon (*p* < 0.05) (Figure 4A–F). Furthermore, O-FMT downregulated the expression of P-p65 and p-IκBα in the NF-κB signaling pathway (Figure 4G–I) of UC mice (*p* < 0.05). Similar to the OLE group, the O-FMT group exhibited a reduction in colonic TJ proteins (ZO-1 and claudin-3) induced by DSS (*p* < 0.05) (Figure 4J–L). Overall, these results suggested that the gut microbiota played a role in the protective effect of OLE against DSS-induced UC in mice.

### 3.4. OLE Altered the Gut Microbiota of Mice with UC

**Figure 5:**
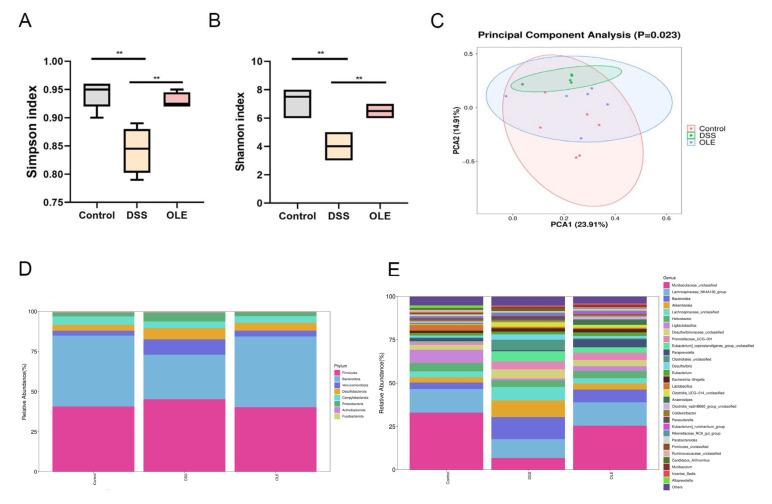
The influences of OLE administration on gut microbiota diversity. (**A**) Simpson diversity index. (**B**) Shannon diversity index. (**C**) Principal coordinate analysis (PCoA) plot illustrating microbial community clustering. Alterations in the intestinal microbial community following DSS exposure are shown at (**D**) the phylum taxonomic level and (**E**) the genus taxonomic level. All data are presented as mean values ± standard deviation (*n* = 6). Statistical significance is denoted as ** *p* < 0.01 when compared with the DSS-treated control group.

The fecal samples were collected from the mice, and the gut microbial composition was analyzed using 16SrRNA sequencing. Firstly, α-diversity was examined. The results revealed a significant increase in α-diversity in OLE-treated mice compared with DSS-treated mice (Figure 5A,B). A PCoA was performed on fecal samples, showing a significant difference in the gut microbial structure between the DSS group and the control group (Figure 5C). Additionally, it was found that DSS treatment significantly reduced the abundance of *Bacteroides*, *Desulfovibrio*, and *Helicobacter* at the gut microbiota level, which, however, was reversed by OLE treatment (Figure 5D). At the genus level, the addition of DSS reduced the abundance of *Lachnospiraceae_NK4A136*, *Lactobacillus*, *Turicibacter*, *Bacteroides*, *Bifidobacterium*, and *Ruminococcus*, but OLE increased the abundance of *Lactobacillus*, *Turicibacter*, *Alistipes*, *Bifidobacterium*, and *Ruminococcus* (Figure 5E). In conclusion, these results suggested that OLE regulated the disorder in the gut microbial structure induced by DSS and contributed to the prevention of DSS-induced UC.

### 3.5. OLE Alleviated UC in Mice by Modulating the Level of Bile Acids in the Gut Microbiota

**Figure 6:**
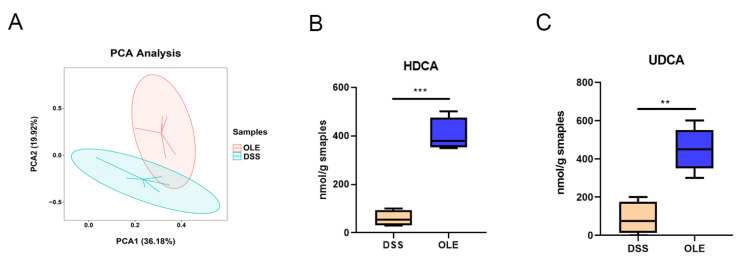
OLE alters the metabolism of mice. (**A**) Principal component analysis. (**B**,**C**) OLE alters representative substances in mouse metabolism (HDCA and UDCA). Each value was expressed as the mean ± SD (*n* = 6). ** *p* < 0.01 and *** *p* < 0.001 vs. DSS.

A change in metabolism is considered to be one of the most important features of UC. In order to study the role of OLE in modulating the intestinal microbiota-related metabolic phenotype involved in DSS-induced UC progression, we performed a metabolomic analysis on serum collected from mice treated with DSS and OLE. The PCA analysis revealed a significant separation between the DSS group and the OLE group (Figure 6A). Therefore, a targeted analysis was performed on the intestinal bile acid metabolism in the DSS and OLE groups using UPLC-MS. The results revealed the relative abundance of major bile acids in the intestines of the mice. Overall, the concentration of primary, secondary, and total bile acids was significantly lower in the DSS group (Figure 6B,C). Specifically, several bile acids with the highest variable importance in projection values, namely, HDCA and UDCA, were significantly reduced in the DSS group compared with the OLE group. These findings indicated the differences in intestinal bile acid metabolism between the DSS and OLE mice.

### 3.6. HDCA Exerted Anti-Inflammatory and Barrier-Protective Effects in Mice with UC

**Figure 7:**
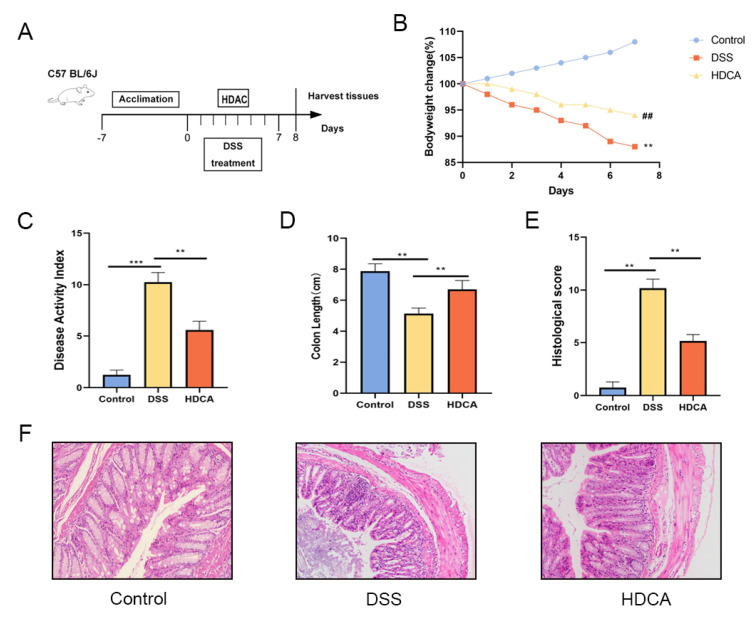
HDCA treatment alleviated DSS-induced colitis in mice. (**A**) Schematic of the experimental design. (**B**) Percentage changes in body weight. (**C**) Disease activity index (DAI) scores. (**D**) Colon length measurements. (**E**) Histopathological scoring results. (**F**) Colonic tissues from each group were prepared for histological analysis, with representative images showing pathological changes across experimental groups. Data are presented as the mean ± standard deviation (*n* = 9). Statistical significance was denoted as ** *p* < 0.01, and *** *p* < 0.001 when compared with the DSS-treated group, ## *p* < 0.01 when OLE compared with the DSS-treated group.

**Figure 8:**
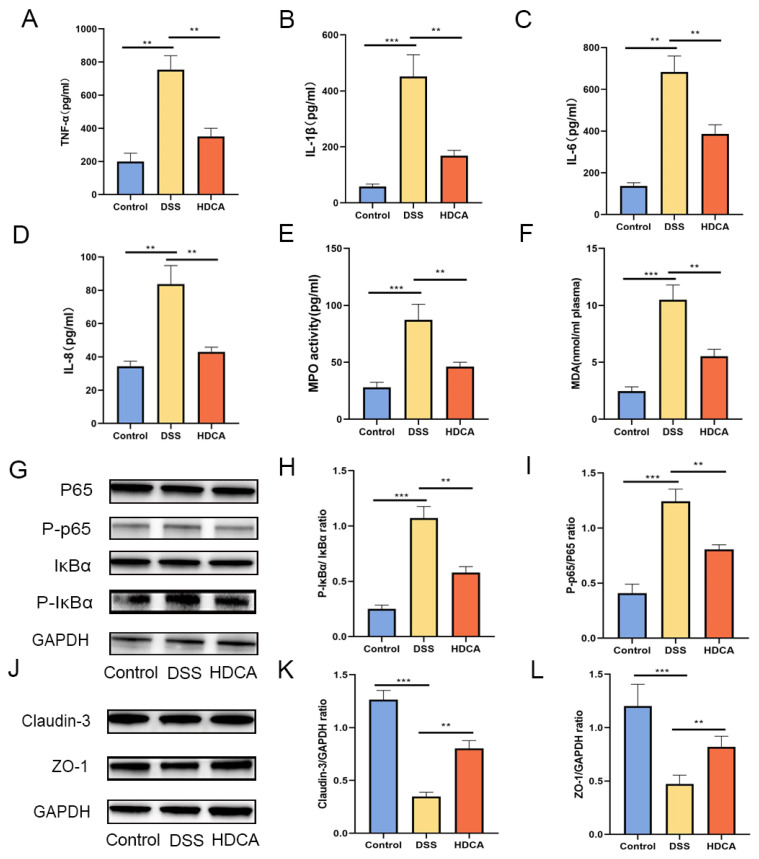
HDCA modulated the expression of pro-inflammatory cytokines, oxidative stress mediators, and intestinal tight junction proteins in colonic tissues. Levels of TNF-α (**A**), IL-1β (**B**), IL-6 (**C**), and IL-8 (**D**)—key pro-inflammatory cytokines—were measured, along with the oxidative markers MPO (**E**) and MDA (**F**). Inhibitory effects on NF-κB signaling (**G**–**I**) were also evaluated, as HDCA exerted protective effects against colitis partly through this pathway. A Western blot analysis was used to assess the protein expression of tight junction components, including claudin-3 and ZO-1, across the three experimental groups (**J**–**L**). Data are presented as the mean ± standard deviation (*n* = 6). Statistical significance was denoted as, ** *p* < 0.01, and *** *p* < 0.001 when compared with the DSS-treated group.

HDCA showed the largest difference in the bile acid content between the OLE and DSS groups, which was associated with an increase in the severity of colonic inflammation in the DSS-treated mice. Based on these findings, relevant experiments were conducted to identify if HDCA could achieve a similar anti-UC effect as OLE (Figure 7A). The results demonstrated that HDCA significantly alleviated DSS-induced colitis, which was proved by changes in body weight, DAI score, and colon length (*p* < 0.05) (Figure 7B–F). Compared with the DSS group, HDCA administration significantly reduced inflammatory cell infiltration and epithelial damage (*p* < 0.05). Additionally, HDCA inhibited the protein expression of pro-inflammatory cytokines (IL-1β, IL-6, and TNF-α) and oxidative stress markers in the colon (Figure 8G–I). At the same time, HDCA downregulated the expression of P-p65 and p-IκBα in the NF-κB signaling pathway of UC mice (Figure 8A–I) (*p* < 0.05). Similar to the OLE group, the HDCA group alleviated the reduction in TJ proteins (ZO-1 and claudin-3) in colitic mice (Figure 8J–L) (*p* < 0.05). Furthermore, HDCA upregulated the protein expression of the bile acid receptor FXR in the colon (Supplementary Figure S1A,B) (*p* < 0.05). In conclusion, the addition of HDCA exhibited similar anti-inflammatory effects to the direct administration of OLE.

## 4. Discussion

The treatment of intestinal inflammation is a complex biological process involving multiple stages, including the repair of damaged intestinal mucosa, the regeneration of intestinal epithelial cells, and, ultimately, the termination of inflammation and restoration of tissue homeostasis [13,14,15,16]. Currently, conventional therapies for UC mainly include aminosalicylates, corticosteroids, and immunosuppressants [17,18,19]. However, these agents often induce severe adverse effects and can even pose life-threatening risks. The relationship between colitis and the gut microbiota has been highlighted in many studies. An imbalance in the gut microbiota can lead to the onset of UC, while UC can also affect the structure and function of the gut microbiota [20]. As revealed in some studies, changing the composition of the gut microbiota can affect the development of UC [21,22]. Therefore, maintaining a healthy gut microbiota could contribute to the prevention and treatment of colitis [23]. The antimicrobial defense mediated by the gut microbiota can be realized through various means, including the production of short-chain fatty acids and the modulation of bile acid metabolism [24]. The DAI is a critical metric for evaluating the health status of UC-model mice [25]. Its scoring system comprehensively quantifies clinical parameters, including weight changes, fecal characteristics, and rectal bleeding, playing a pivotal role in the pathological assessment of UC models [26]. This study showed that oral administration of OLE significantly improved clinical symptoms in DSS-induced colitis mice: compared with the DSS-model group, OLE-treated mice exhibited reduced body weight loss, alleviated colon length shortening, and attenuated increases in DAI scores, indicating that OLE repairs DSS-induced intestinal damage.

In oxidative-stress-injury-related assays, the DSS-model group displayed significantly elevated MPO activity and MDA content in colon tissues alongside significantly decreased activities of antioxidant enzymes. Notably, OLE intervention reversed these oxidative-stress-related abnormalities, suggesting that OLE protects against DSS-induced colitis by mitigating oxidative damage in colon tissues. Concurrently, this study revealed that OLE administration suppressed pro-inflammatory cytokines via the NF-κB signaling pathway, increased TJ protein levels in DSS-treated mice, and maintained intestinal environmental stability—findings that corroborate the therapeutic effects of OLE on DSS-induced UC [27]. To validate the role of the gut microbiota in OLE-mediated UC treatment, microbiota from OLE-treated mice were transferred into UC mice. The results showed that OLE-FMT reduced UC severity, indicating that OLE exerts its therapeutic effects—at least, in part—by modulating the gut microbiota. Notably, OLE-FMT successfully alleviated acute colitis with significantly superior efficacy to CON-FMT, highlighting the dominant role of OLE-mediated gut microbial changes in colitis remission. Collectively, these results demonstrate that OLE-modulated gut microbiota are critical for UC remission, and we hypothesized that OLE improves colitis by increasing the abundance of low-abundant beneficial microbes in healthy controls [28]. To elucidate the mechanisms, 16SrRNA sequencing was performed, revealing that OLE restored the intestinal barrier by increasing *Lactobacillus* and decreasing *Proteobacteria* abundances.

Research indicates that bile acids are crucial in UC pathogenesis, with metabolic dysregulation linked to gut microbiota imbalances, mucosal immune dysfunction, and intestinal barrier damage [29]. As digestive and signaling molecules, UC patients exhibit elevated primary bile acids and reduced secondary bile acids. This imbalance inhibits beneficial bacterial colonization while promoting pathogen growth. Simultaneously, it regulates immune homeostasis through nuclear receptor FXR and membrane receptor TGR5. FXR activation suppresses the NF-κB pathway and strengthens the intestinal barrier, but UC patients show downregulated intestinal FXR expression, disabling anti-inflammatory protection [30]. The TGR5 pathway mediates anti-inflammatory cytokine secretion by lamina propria immune cells and inhibits Th17 cell differentiation [31]. Excess primary bile acids disrupt the intestinal mucus layer and induce epithelial cell apoptosis, exacerbating mucosal injury [32]. Clinically, fecal bile acid profiles and serum taurine-conjugated bile acid levels serve as potential markers for UC activity and severity, while strategies targeting the microbiota–bile-acid axis and activation of FXR/TGR5 pathways offer new therapeutic directions [33,34,35].

In subsequent experiments, targeted bile acid metabolomics analyses revealed that OLE significantly reshaped the bile acid profile in UC-model mice. Supplementation with the most differentially expressed bile acid metabolite, HDCA, in DSS-induced UC mice showed that HDCA downregulated pro-inflammatory cytokines via inhibition of the NF-κB pathway and enhanced TJ protein levels to maintain intestinal mucosal barrier integrity, thereby restoring gut microenvironmental homeostasis. Emerging studies have confirmed that FXR plays a key regulatory role in chronic intestinal inflammation, with its mucosal homeostasis-mediated mechanisms often impaired by inflammatory responses [36,37]. Clinical data show significant FXR functional defects in UC- and colitis-associated colorectal cancer patients, while inflammation-induced epithelial abnormalities disrupt FXR signaling and reshape bile acid profiles. This study found that HDCA activated the FXR signaling pathway in UC mice, suppressing pro-inflammatory cytokine secretion and mediating the pathological process of intestinal inflammation. In conclusion, the therapeutic effects of OLE on UC likely involve a complex regulatory network of multiple pathways.

In summary, the experiments in this study revealed that these OLE compounds could be employed in the treatment of DSS-induced colitis by improving abnormal inflammatory responses and microbial dysbiosis. Additionally, OLE increased microbial-derived bile acid metabolites, which induced the inhibition of the NF-ĸB signaling pathway and protected the intestinal mucosal barrier. These findings suggest that OLE could be an alternative option for the treatment of UC and other intestinal diseases.

## Data Availability

All related data and methods are presented in this paper. Additional inquiries should be addressed to the corresponding author.

## Supplementary Materials

The following supporting information can be downloaded at https://www.mdpi.com/article/10.3390/foods14111863/s1. Figure S1: HDCA modulated the expression of proteins in colonic tissues (A,B). Data are presented as the mean ± standard deviation (*n* = 6). Statistical significance was denoted as * *p* < 0.05, and ** *p* < 0.01 when compared with the DSS-treated group.
