# Supplementary material for: Oleuropein Regulates Bile Acid Metabolism via Modulating the Gut Microbiota, Thereby Alleviating DSS-Induced Ulcerative Colitis in Mice
Source: Foods. 2025 May 23;14(11):1863. doi: 10.3390/foods14111863 (PMC12155123; doi:10.3390/foods14111863)
Supplement: Supplementary file 1 [file foods-14-01863-s001.zip › foods-3574715-supplementary.pdf]

Article

Oleuropein Regulates Bile Acid Metabolism via Modulating the Gut Microbiota, Thereby Alleviating DSS-Induced Ulcerative Colitis in Mice

Rongxin Zang [1,2], Rui Zhou [1], Yaodong Li [1], Zhouliang Liu [1], Huihao Wu [3], Liping Lu [1] and Hongwei Xu [1,2,*]

1 College of Life Science and Engineering, Northwest Minzu University, Lanzhou 730100, China; rxzang2000@163.com (R.Z.); zhour1222@163.com (R.Z.); lyaodong@163.com (Y.L.); a134063518367@163.com (Z.L.); 901030@126.com (L.L.)
2 Engineering Research Center of Key Technology and Industrialization of Cell-Based Vaccine, Ministry of Education, Lanzhou 730030, China
3 Key Laboratory of Biotechnology and Bioengineering of State Ethnic Affairs Commission, Northwest Minzu University, Lanzhou 730030, China; wuhuihao99@163.com
* Correspondence: xuhongwei@xbmu.edu.cn

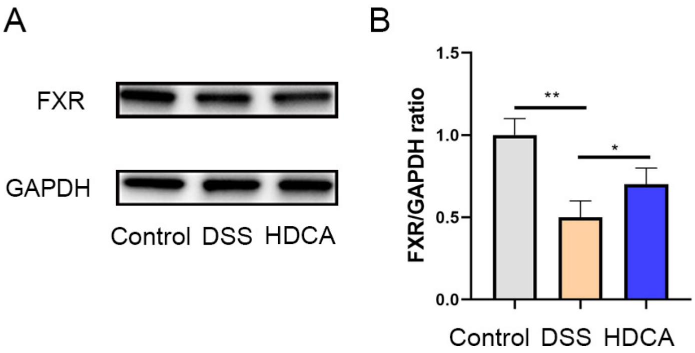

Figure S1. (**A**,**B**) HDCA modulated the expression of proteins in colonic tissues (**A**,**B**) Data are presented as the mean ± standard deviation (n = 6). Statistical significance was denoted as * $p < 0.05$ and ** $p < 0.01$ when compared to the DSS-treated group.
